# Supplementary material for: WordCommentsAnalyzer: A windows software tool for qualitative research
Source: F1000Res. 2018 Sep 4;7:536. Originally published 2018 May 3. [Version 2] doi: 10.12688/f1000research.14819.2 (PMC6137411; doi:10.12688/f1000research.14819.2)
Supplement: Supplementary file 3 [file f1000research-7-17614-s0002.tgz › b2a79496-4ada-47c4-9682-a99ce867836d.zip!/Eight Iranian Health Journals/Journal of Research in Rehabilitation Sciences _17.docx]

© Journal of Research in Rehabilitation Sciences . This work is licensed under CC BY-NC 4.0 (https://creativecommons.org/licenses/by-nc/4.0/).

Volume & Issue: ویژه نامه توانبخشی، دوره 10، شماره 8، 1393

Title: اثربخشی تمرین بازداری شناختی بر انعطاف پذیری ذهنی دانش آموزان با ناتوانی یادگیری

http://jrrs.mui.ac.ir/index.php/jrrs/article/view/2030

مقدمه: کارکردهای اجرایی از قبیل بازداری و حافظه فعال برای بسیاری از رفتارهای انسان اهمیت دارند. از اینرو بر مداخلاتی که منجر به بهبود کارکردهای اجرایی می­شوند تأکید شده است. هدف این پژوهش بررسی تأثیر تمرین بازداری شناختی بر عملکرد انعطاف پذیری ذهنی دانش­آموزان با ناتوانی یادگیری بود. مواد و روش­ها: طرح این پژوهش از نوع پیش آزمون پس آزمون با گروه کنترل بود. 30 دانش­آموز به صورت نمونه­گیری در دسترس انتخاب شدند (15 نفر در گروه آزمایش و 15 نفر در گروه گواه) و گروه آزمایش به مدت 10 جلسه تحت مداخله تمرین بازداری شناختی قرار گرفت. همه شرکت کنندگان در مرحله پیش آزمون و پس آزمون توسط آزمون مرتب کردن کارت ویسکانسین مورد سنجش قرار گرفتند. نتایج به دست آمده با استفاده از روش تحلیل کوواریانس چندمتغیره در نرم افزار SPSS نسخه 19 (version 19, SPSS Inc., Chicago, IL) مورد تحلیل قرار گرفت. یافته­ها: یافته­ها نشان داد که بین گروه آزمایش و کنترل در تعداد طبقات تفاوت معنادار وجود دارد (001/0= P) همچنین بین گروه آزمایش و کنترل تفاوت معناداری در خطای درجاماندگی وجود داشت (05/0= P). نتیجه گیری: مطابق با نتایج به دست آمده، تمرین بازداری شناختی  بر عملکرد انعطاف پذیری ذهنی تأثیر مثبت دارد.

Title: تاثیر انواع کفی و کفش بر تعادل سالمندان: مطالعه مروری

http://jrrs.mui.ac.ir/index.php/jrrs/article/view/1731

چکیده افراد سالمند به دلیل کاهش در تواناییهای حسی و حرکتی دچار  کاهش تعادل نیز می شوند. مشکلات تعادل در این افراد می تواند منتج به زمین خوردن و به دنبال آن آسیب ها جدی تر و شاید مرگ نیز شود. زمین خوردن به دلیل عواقب وخیم باعث افزایش هزینه های درمانی در سیستم بهداشت و درمان می گردد. کفش و کفی به عنوان یک واسط بین کف پا و سطح زمین می تواند بر کیفیت بازخوردهای حسی و تعادل افراد تاثیر گذار باشد. از این منظر بررسی نقش انواع کفی ها و کفش ها بر تعادل افراد سالمند می تواند در پیشگیری از زمین خوردن این افراد مورد توجه قرار گیرد. هدف از این مرور متون بررسی تحقیقات انجام شده در حیطه مداخلات مختلف کفی و کفش بر تعادل افراد سالمند بوده است.  در این مطالعه مروری، جستجوی نظام­مندی درپایگاه­های الکترونیکی مختلف انجام شد. مقالات مرتبط از طریق جستجوی واژگان کلیدی (شامل ترکیبات و مترادفات مختلف از جمله سالمندی، تعادل، کفی و کفش) استخراج شد.  پس از غربالگری، نتایج ارایه شده مقالات منتخب از نظر موضوع مورد بررسی قرار گرفتند. به طور کلی مداخله کفی و کفش از سه طریق می تواند بر تعادل این افراد تاثیر بگذارد: اول، توسط تقویت بازخورد حسی کف پا (مانند کفی های لرزاننده و یا دارای برجستگی)، دوم بوسیله تغییر در فشار کف پایی (مانند کفی های ساپورت قوس) و سوم توسط تغییر در راستای مچ پا (مانند تغییر ارتفاع پاشنه). نتایج این بررسی نشان داد که کاهش عوامل حسی به دلیل پدیده­ کهنسالی در افراد سالمند منجر به شرایط خطرساز مانند کاهش تعادل و زمین خوردن ­شود. همچنین بر اساس شواهد به نظر می رسد که بتوان به ­وسیله مداخلات مناسب و مختلف کفی و کفش، در کاهش بی ثباتی و عوارض آن در این دوره از زندگی موثر بود.

Title: نقش پلاستیسیتی کراس مدال در سیستم شنوایی و بینایی

http://jrrs.mui.ac.ir/index.php/jrrs/article/view/1608

مقدمه: نروپلاستی سیتی توانایی سیستم عصبی در ایجاد تغییر و ترمیم پس از وقوع آسیب است. وقتی یکی از مدالیته های حسی حذف می شود در رفتار سایر مدالیته ها و عملکرد کورتیکال آن ها تغییراتی ایجاد می شود، به گونه ای که پردازش این مدالیته ها علاوه بر کورتکس اختصاصی ِ مربوط به پردازش آن، به سایر مناطق مغز که ورودی حس خود را دریافت نمی کنند نیز گسترش می یابد و برای آن منطقه از مغز که از ورودی حسی خود محروم شده است فرآیندی جبرانی صورت می پذیرد. به این پدیده پلاستی سیتی کراس مدال گفته میشود. این پژوهش به بررسی جوانب مختلف پلاستی سیتی کراس مدال بین سیستم های شنوایی و بینایی و آخرین یافته های علمی در این زمینه میپردازد. مواد و روش ها: مقالات موجود در پایگاه های استنادی Pubmed, Google Scholar, Science Direct, با استفاده از کلیدواژه های Neuro- plasticity and Auditory, Neuro-plasticity and Visual, Neuro-plasticity  در فواصل سالهای 1980 تا 2013 مورد بررسی قرار گرفت. یافته ها: پس از بررسی چکیده و محتوای مقالات، از مجموع 95 مقاله به دست آمده، تعداد 70 مقاله با محتوای مورد نظر به دست آمده و به طور کامل بررسی شد. نتیجه گیری: درجات پلاستی سیتی کرال مدال به سن وقوع محرومیت در آن حس خاص وابسته است. در افراد نابینا  حواس شنوایی و سوماتوسنسوری به اشغال فضاهای اکسی پیتال می پردازند و موجب تقویت فعالیت هایی نظیر شنیدن، بازیابی اطلاعات از حافظه، مکان یابی های فضایی، درک موسیقی و درک خط بریل میشوند. در افراد ناشنوا نیز حواس بینایی و سوماتوسنسوری بخش هایی از لوب تمپورال را به خود اختصاص داده و و فعالیت هایی نظیر لوکالیزاسیون بینایی، پردازش محرکات بینایی محیطی، تقویت حس لامسه و پردازش حرکت را تقویت میکنند.

Title: بررسی یادگیری حرکتی در بیماران مبتلا به سکته¬ی مغزی یک طرفه: مروری نظام مند بر مطالعات گذشته

http://jrrs.mui.ac.ir/index.php/jrrs/article/view/1978

مقدمه : هدف از انجام مطالعه ی حاضر مرور نظام مند بر مطالعات گذشته ای است که به بررسی یادگیری حرکتی در بیماران سکته¬ی مغزی یک طرفه پرداخته اند. مواد و روش ها : جست جوی مطالعات انجام شده در فاصله زمانی 1995 تا2012 در منابع اطلاعاتی Elsevier, Science Direct, ProQuest ,google scholar, PubMed انجام شد کلید واژه هایهای hemiplegia, stroke ,learning, motor learning, recovery, functional recovery بعنوان کلید واژه ها مورد استفاده قرار گرفتند. یافته ها : در کل 10 مقاله که دارای معیارهای ورود بودند انتخاب شدند. تفاوت هایی میان مقالات از نظر متدولوژی، شدت و محل ضایعه، تعداد نمونه، روش کار مشاهده گردید. نتیجه گیری : بررسی مرور مطالعات نشان داد توانایی یادگیری حرکتی تلویحی بیماران سکته¬ی مغزی یک طرفه در صورتی که وظایف حرکتی مربوطه، نوع فیدبک و آموزش های صریح به بیمار طوری باشد که نیاز حافظه ی حرکتی را بالا نبرد و یا شدت سکته¬ی مغزی پایین باشد، همچنان حفظ می شود.

Title: بررسی تاثیر انواع روش های درمانی در بیماران دارای استئوآرتریت کمپارتمان داخلی زانو: مروری بر مقالات

http://jrrs.mui.ac.ir/index.php/jrrs/article/view/1716

پیش‌زمینه و هدف: استئوآرتریت زانو یكی از مشكلات اسكلتی-عضلانی شایع در بالغین است كه باعث ایجاد درد، بیحركتی و ناتوانی در افراد مبتلا می‌گردد و شیوع آن در زنان بیشتر از مردان است. در استئوآرتریت زانو درگیری كمپارتمان داخلی زانو تقریباً 10 برابر بیشتر از كمپارتمان خارجی است و در نتیجه اغلب باعث ایجاد دفورمیتی واروس در افراد مبتلا می شود. درمان استئوآرتریت زانو بیشتر با هدف كاهش درد، بهبود عملكرد فیزیكی و كاهش روند پیشرفت بیماری از طریق اصلاح راستای زانو وکاهش گشتاور وروس انجام می‌پذیرد، که بر همین اساس درمان‌ها به طور کلی در دو دسته قرار می گیرند که شامل: استفاده از روش‌های جراحی و درمان‌های غیر جراحی و نگهدارنده (مانند ارتوزهای زانو و کفی طبی با لبه خارجی، استفاده از تکنیک های فیزیوتراپی، ورزش و کاهش وزن در افراد چاق و آموزش اصول مراقبت از مفصل) می‌باشد. هدف از این مقاله مروری بررسی تاثیر انواع روش‌های درمانی نگهدارنده در بیماران دارای استئوآرتریت کمپارتمان داخلی زانو است. مواد و روش کار: پس از جستجو در پایگاههای اطلاعاتی Science Direct ,PubMed وGoogle scholar، مقالات مرتبط با موضوع از سال 1973 الی 2012 گردآوری شد. کیفیت مقالات با استفاده از ابزار Black and Down مورد ارزیابی قرار گرفت.کلمات کلیدی زیر برای جستجو استفاده گردید: OAknee ، Knee orthosis، Valgus brace،Weight Loss ،Gait ،Joint loading  ،Obesity ، Rehabilitation، Physical Therapy،.Lat wedge insole یافته‌ها: بر اساس کلمات کلیدی استفاده شده،60 مقاله مرتبط گردآوری شد و از بین آنها 24 مقاله با توجه به معیارهای انتخاب برای آنالیز نهایی پذیرفته شدند. ارزیابی کیفی مقالات با استفاده از پرسشنامه Downs and Black tool انجام شد و نمره‌ی 4 معیار این پرسشنامه یعنی گزارش دهی، روایی خارجی، روایی داخلی (بایاس) و روایی داخلی(کانفوندینگ) به ترتیب 9تا2، 1 تا 3، 2 تا 5 و 2 تا 5 بود. نتیجه گیری: پس از گردآوری مقالات و انجام بررسی های نهایی و جمع بندی کلی مباحث، در بین روش‌های درمانی غیر دارویی و غیر جراحی نگهدارنده‌ای که ذکر شد، به نظر می رسد که همگی این روشها نتایج مطلوبی بر کاهش گشتاور و درد و ناتوانی داشته باشند ولی با توجه به اینکه حجم مقالات قابل ملاحظه نیست برای ارائه یک نتیجه‌ی کلی با قطعیت کامل نیاز به پژوهش‌های بیشتر می باشد.

Title: مروری بر مطالعات آکوستیک ماهیت خطاهای گفتاری در افراد مبتلا به آفازی بروکا

http://jrrs.mui.ac.ir/index.php/jrrs/article/view/1710

مقدمه: آفازی بروکا یکی از انواع آفازی های ناروان است و تولید غیر دقیق صداهای گفتاری از ویژگی های اصلی آن میباشد. بررسی های مختلف نشان داده است که بیماران مبتلا به آفازی برو کا نقایصی دارند که ساختار صدا ها را در خروجی گفتار آن ها تحت تاثیر قرار می دهد اما کمتر مشخص شده است که ماهیت این خطاها یک اختلال در انتخاب و برنامه ریزی است یا اختلال در اجرای تولیدی. تعیین ماهیت خطاهای گفتار در این بیماران در تشخیص مکانیسم های  اساسی مختل و موثر در ایجاد این خطاها بسیار ضروری و مهم می باشد. هدف این مطالعه، مروری بر مطالعاتی است که ماهیت خطاها در بیماران آفازی بروکا را به شیوه آکوستیک بررسی نموده اند. مواد و روش ها: درمطالعه مروری- روایتی حاضر ، با استفاده از کلید واژه های  "پارامترهای آکوستیک ، ماهیت خطاها  ، آفازی بروکا ،کنترل حرکتی گفتار، خطاهای گفتاری" پایگاه های اطلاعاتی sience direct، pubmed ، web of knowledge ،google scholar را جستجو نمودیم . یافته ها: نقایصی در زمینه کنترل حرکتی گفتار در افراد مبتلا به آفازی بروکا وجود دارد که این نقایص شامل مشکلات در زمانبندی ، هماهنگی حرکات تولیدی و کنترل حنجره ای می باشد و نتیجه نقص در هماهنگی و یکپارچه سازی حرکات تولیدی و اجرای تولیدی مورد نیاز برای تولید سگمنت هدف می باشد. نتیجه گیری: پژوهش های اخیر از طریق بررسی آکوستیک الگوهای تولید گفتار نشان داده است ویژگی اصلی آسیب گفتاری در افراد مبتلا به آفازی بروکا یک اختلال آوایی شدید مربوط به اجرای تولیدی قطعات گفتاری انتخاب شده و برنامه ریزی شده می باشد .

Title: ویژه یاری: حلقه نادید هندلینگ کودک فلج مغزی: نامه ای به سردبیر

http://jrrs.mui.ac.ir/index.php/jrrs/article/view/2165

ویژه یاری، مراقبت ویژه و خاصی است در حیطه کاره­های روزمره زندگی کودک فلج مغزی، که توسط مراقب که عمدتا مادر است صورت می­گیرد. ناآگاهی متقابلی از طرف مادران و درمانگران، نسبت به ویژه یاری وجود دارد. یعنی نه تنها مادران، آگاهی لازم نسبت به ویژه یاری را ندارد بلکه عملا هم تجربه ای در این زمینه ندارد و ویژه­یاری بعنوان حلقه نادید هندلینگ، موجب سختی، تنیدگی و افزایش بار مراقبتی در مراقبان گردیده است. بنظر می­رسد از رهگذر برنامه­های آموزشی مراقبتی به والدین و درمانگران، برگزاری کارگاه­های آموزشی و آشنايي عملي و علمي با نحوه­ی مراقبت از کودک فلج مغزی می توان به افزایش سطح آگاهی و ارتقا تجربه آنها در مورد ویژه یاری همت گماشت.
